# Green synthesis characterization and antimicrobial activity against *Staphylococcus aureus* of silver nanoparticles using extracts of neem, onion and tomato

**DOI:** 10.1039/c9ra01407a

**Published:** 2019-05-30

**Authors:** Kishore Chand, M. Ishaque Abro, Umair Aftab, Ahmer Hussain Shah, Muhammad Nazim Lakhan, Dianxue Cao, Ghazanfar Mehdi, Abdalla Mohamed Ali Mohamed

**Affiliations:** Key Laboratory of Superlight Material and Surface Technology, Ministry of Education, Harbin Engineering University 150001 China caodianxue@hrbeu.edu.cn; Department of Metallurgy & Materials Engineering, Mehran University of Engineering & Technology Jamshoro 76062 Pakistan; Department of Textile Engineering, Baluchistan University of Information Technology, Engineering and Management Sciences Quetta 87300 Pakistan; College of Energy and Power Engineering, Harbin Engineering University 150001 China

## Abstract

Recently, it has been shown that silver nanoparticles (AgNPs) exhibit great potential for different applications, including food storage, cosmetic products, electronic components, biosensor materials, cryogenics, dental materials and especially for drug-delivery activities. In this study, we synthesized AgNPs with neem extract (NE) alone and mixed plant extracts of neem, onion and tomato (NOT) as a combined reducing and stabilizing agent by a green synthesis method at different pHs. The synthesized products were characterized by ultraviolet-visible spectroscopy (UV-vis), X-ray diffraction (XRD), dynamic light scattering (DLS), atomic force microscopy (AFM), scanning electron microscopy (SEM) and transmission electron microscopy (TEM). The antibacterial effects of the synthesized products were studied by the Kirby disk diffusion method. It was confirmed that the AgNPs work effectively as a drug material against Gram-positive bacteria *Staphylococcus aureus* in nutrient agar. In addition, it was seen that the reducing and stabilizing agent NOT could work effectively with six medicines with a different nature at the maximum addition of 15 μg. However, the synthesized product with NE alone only worked for four of the medicines. Therefore, it was evident that the AgNPs synthesized with NOT extract were more susceptible to the Gram-positive bacteria *Staphylococcus aureus*. We believe that this new route for synthesizing AgNPs with NOT extract could be more beneficial in comparison to NE alone for improved antibacterial properties in drug-delivery applications.

## Abstract 2

The silver nanoparticles were synthesized from the neem leaves, onions, and tomato extracts, and the antibacterial activity of the particles was studied.

## Graphical abstract

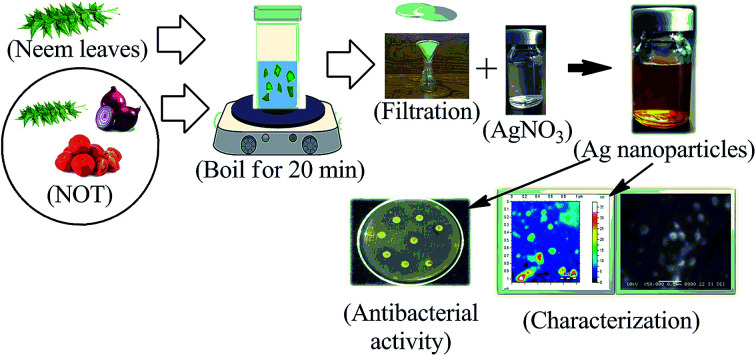

## 1. Introduction

Nanotechnology is an emerging field of interest for medical chemistry, materials science, atomic physics and many other fields. More specifically, nanoparticles (NPs) are considered as particles having a size less than 100 nm in at least one of three promising dimensions.^1^ The physical, chemical and biological properties of these particles are altered in a number of basic ways from atoms, molecules and the bulk materials. NPs have different chemical natures, such as polymers, ceramics, organics, carbon, silicates, biomolecules, metals, metal oxide and non-oxides. NPs can be produced in various different structures, like spheres, triangular, cylinders, platelets and tubes. The use of NPs in different applications is dependent on many parameters, including their size, geometry and morphology; however, the most important one is size, especially in drug-delivery systems.

The antibacterial activity of metal nanoparticles is most promising in the field of drug delivery, where they are considered the most interesting materials in clinical research due to the increasing microbial resistance to metal ions, antibiotics and the resistance development of various strains.^2^ Silver nanoparticles (AgNPs) are quite substantial because of their large surface area relative to their volume. Due to their outstanding antibacterial properties, AgNPs have been applied in various different applications, such as antibacterial applications, fibre-reinforced composites, food storage, drug delivery, cosmetic products, gas sensors, superconducting materials, cryogenic electronic components, coatings and other environmental applications.^3,4^

Neem belongs to the Meliaceae family and has been known for more than 200 years as one of the most popular medicinal plants, having a broad spectrum of biological activity. Each and every part of neem is used as a traditional medicine for various diseases.^8–10^

The major advantages of using extracts of neem leaves are that it is a commonly available medicinal plant and the antimicrobial activity in the green synthesis of AgNPs may be enhanced by capping with neem leave extracts.^5–7^ The use of mixed plant extracts of neem, onion and tomato (NOT) is a very new field, for which no one has reported results before. It is also very important because the mixture of three plants and also the combination of different compounds play a vital role in synthesizing silver nanoparticles that perform with better antibacterial results because of the way the different compounds react with each other. It may be also used in dye degradation and many other applications.

In order to synthesize and stabilize AgNPs, numerous approaches have been used, including mechanical milling, sol–gel techniques, precipitation, hydrothermal, microwave heating, electrochemical and biological synthesis. All these approaches can prepare particles with a distinct surface area, shape, size and size distribution, but green synthesis is the most popular emerging field because of it being typically low cost and less time consuming and eco-friendly.^11,12^ Another advantage includes that a variety of shapes can be synthesized by varying the proportions of the extracts.^13^Table 1 shows some literature date for the extracts used to date for the synthesis along with the specific size, shape obtained and applications.

**Table 1:** Literature work about the green synthesis of AgNPs and Au by different researchers using different plant extracts

Plant extract	Nanoparticles	Results	References
*Aloe vera*	Ag/Au, spherical, 5–50 nm	Highly active against Gram-positive *S Aureus* and Gram-negative *E. coli*	22
*Safeda* leaves	Ag/Au, 50–150 nm	Good antimicrobial activity against waterborne pathogens like *E. coli* and *Vibrio cholerae*	23
*Garcinia mangostana*	Ag, 35 nm	Excellent bactericidal activity in Gram-negative and Gram-positive bacteria	24
*Pyrus* sp. (pear fruit extract)	Au, hexagonal triangular, 200–500 nm	Particles could be internalized through endocytosis by MCF-7 breast cancer cells	25
*Nelumbo Nucifera* (Lotus)	Ag, Au, triangular, spherical, 25–80 nm	These nanoparticles are active against *E. coli*	26
*Ocimum sanctum* leaves	Ag, spherical, 4–30 nm	High antimicrobial action against Gram-positives *Streptococcus aureus* and Gram-negative (*E. coli*)	27
*Mentha piperita* (peppermint)	Ag, spherical, 5–150 nm	Adulticidal and larvicidal against the hematophagous fly *Hippobosca* maculate and the sheep louse *Bovicolaovi*	28
Jambul seeds	Ag/Au, spherical, 29–92 nm	The antimicrobial activity of these nanoparticles was shown to prevent positive and negative bacteria	25
Banana peels	Ag, spherical, 20 nm	These nanoparticles displayed antifungal activity against the yeasts *C. albicans* and Candida and antibacterial activity against *E. coli*, and *Enterobacter aerogenes*	29
Neem extract, lemon juice	Ag/Au, spherical, 29–92 nm	NPs are very effective against Gram-negative and Gram-positive bacteria	7
Neem gum	Ag, spherical, 30–60 nm Au, spherical, 50–250 nm	Gold and silver nanoparticles have a wide range of antimicrobial activity against animal and human pathogens	30
*Heliotropiumcrispum*	Ag, spherical, 42–120 nm	AgNPs showed good bacterial strain destruction against Gram-negative *Pseudomonas aeruginosa* (PA) and *Acinetobacter baumanii* (AB) and against Gram-positive multiple drug resistant *Staphylococcus aureus* (MRSA)	31
*Diospyros paniculata*	Ag, spherical, 17 nm	Nanoparticles showed excellent performance against Gram-negative and Gram-positive bacteria	32
*Anethum graveolens*	Ag, spherical 35 nm	These AgNPs could be effective as nano-drug carriers in a special category without having an effect on the parasites or host cells, but with an unknown mechanism for enhancing drug availability	33
*Sapindus emarginatus pericarp*	Ag, spherical, 5–20 nm	Silver nanoparticles have remarkable antibacterial activity against many species, such as *Staphylococcus aureus*, *Escherichia coli*, *Bacillus*, *subtilis* and *Proteus mirabilis*	34
*Trachyspermumammi* (Ajwain)	Ag, 12.74 nm	Show highest catalytic activity for the conversion of *p*-nitrophenol to *p*-aminophenol in an excess of NaBH_4_	35
*Madhuca longifolia* flower	Ag, spherical, 30–50 nm	Silver nanoparticles were used in therapeutics (medical applications)	15
*Psidiumguajava* L. leaf	Ag, spherical, 25–35 nm	Nanoparticles were used for medical and cosmetic applications	6
*Berberis* vulgaris leaf and root aqueous extracts	Ag, spherical, 30–70 nm	Compared to others, these nanoparticles have more antibacterial activities and were also tested against *Staphylococcus aureus* and *Escherichia coli*	36
*Ampelocissus latifolia* root	Ag, spherical, 35–45 nm	NPs showed much better antibacterial activity towards Gram-negative and Gram-positive bacteria	37
*Enicostemma axillar* (Lam.) leaf	Ag, spherical, 15–20 nm	Nanoparticles prepared from the plant have more applications in the biomedical field and also have many benefits, such as effectiveness, compatibility for biomedical and pharmaceutical applications, like antifungal and antibacterial, as well as good for large-scale marketable production	38
*Fritillaria* flower	Ag, spherical, 5–10 nm	These nanoparticles were used in medical applications	39
*Fenugreek* seeds	Ag, spherical, 17 nm	NPs were used as an antibacterial agent for *Staphylococcus aureus* and *E. coli*	40
*Melissa officinalis* leaf	Ag, spherical, 12 nm	Silver nanoparticles prepared from *Melissa officinalis* leaf extract provided an efficient and functional methodology to obtain well-dispersed and antimicrobial NPs that provided better results against *E. coli* and *S. aureus* bacteria	41
Saffron (*Crocus sativus* L.)	Ag, spherical, 12–20 nm	The silver nanoparticles showed inhibiting activity against *Pseudomonas aeruginosa*, *Klebsiella pneumonia*, *Escherichia coli* and *Bacillus subtilis*	42
*Azadirachtaindica* aqueous leaf	Ag, spherical, 34 nm	The silver nanoparticles showed antibacterial activities against both Gram-positive and Gram-negative microorganisms	43
*Givotiamoluccana* leaf	Ag, crystalline nature, 30–40 nm	Nanoparticles played an active role in antimicrobial activity against pathogenic bacteria; also used for commercial appliances and other medical and electronic applications as well as cancer treatment, drug delivery, and sensors	44
Turmeric	Ag, triangular, ellipsoidal, decahedral, 5–35 nm	Nanoparticles synthesized with turmeric extract showed extraordinary and proficient antimicrobial activities against two food-borne pathogens (*Listeria monocytogenes* and *Escherichia coli*)	45
Green tea	Ag, 2.17 nm, crystalline	PEG-AgNPs and AgNPs and showed powerful antibacterial effects against several pathogenic Gram-negative and Gram-positive bacteria	46
*M. balbisiana*, *A. indica* and *O. tenuiflorm*	Ag, 14.5–9.10 and 11.0 nm	AgNPs were used against *K. pneumoniae*, *S. aureus*, *E. coli*, and *B. subtlis* and also could be used in different fields, like medical, food and cosmetics	47
Market vegetable waste	Ag, triangular, spherical, 10–90 nm	Nanoparticles showed a positive effect against Gram-positive bacteria (*Staphylococcus* sp.) and Gram-negative (*Klebsiella* sp.)	48

The emergence of antibiotic-resistant strains of *Staphylococcus aureus* (SA), such as Methicillin-resistant; is a worldwide problem in clinical medicine. The main diseases and effects of SA include skin problems, infertility in women, bone and joint infections, urinary tract infections and many other diseases. A lot of research and development has already been performed on it, but still there is a need for developing a more powerful drug against SA.^14,15^ In medicinal applications, AgNPs have prime importance as they are considered a candidate drug-delivery substance. The principle mechanism is based on the penetration of AgNPs in the cell wall and distresses the cell respiration. AgNPs also enter into the cell wall of bacteria or microorganism and destruct the cell by the combined action of sulfur and phosphorus compounds, such as proteins and deoxyribonucleic acid. The antibacterial potential of AgNPs are due to the clemency of Ag^+^ ions from nanoparticles.^16–18^ The bactericidal activity of AgNPs depends not only on their size but also on the pH, salt concentration and the medium in which it is diffused.^19^ Nano-silver's unique physical and chemical properties make it highly bioactive such that it can react with cells, micro-organisms and macro-organisms.^14,20,21^

The present work is based on a green synthesis of AgNPs using extracts of neem leaves (NE) alone and a mixture of neem, onion and tomato (NOT) at different pHs: pH 5, pH 7 and pH 9. The synthesized AgNPs were characterized by different techniques, such as UV-vis, XRD, Zetasizer, AFM, SEM and TEM. Furthermore, the antimicrobial activity of the synthesized AgNPs against SA for seven different classes of antibiotics also fell under the scope of this study. According to the best of the knowledge of the authors, this is the first study to report the green synthesis and antimicrobial activity of AgNPs conducted using extracts of several mixtures at different pH levels.

## 2. Experimental

### 2.1 Materials

Silver nitrate (AgNO_3_) was purchased from Merck Company (Germany). Fresh neem leaves, onion and tomato were purchased from the local vegetable market. Nutrient agar was purchased from Oxoid (UK). Acetone (99%), ethanol (98%), sodium hydroxide (NaOH, 99%) and hydrochloric acid (HCl) were purchased from Aladdin Chemicals China. Distilled water was used throughout the study where necessary.

### 2.2 Preparation of silver nanoparticles

The green synthesis of AgNPs was carried out according to an earlier reported method.^49^ Garden-fresh neem leaves, tomatoes and onions (20 g of each) were cut into smaller pieces and washed with distilled water thoroughly (three times). The tomato, onion and neem leaves were separately mixed in a beaker with 200 ml of distilled water and boiled for 20 min. After boiling, the solution was cooled down for 10 min and double filtered by Whatman paper 1. 1 mM solution of silver nitrate was prepared by dissolving 16.987 g of silver nitrate in 1 L of distilled water in a beaker and kept in the airtight bottle until further use. NE (6 ml) and NOT (6 ml) were mixed using a mechanical stirrer for 10 to 15 min with a 10 ml solution of silver nitrate in the beaker at different pH values (5, 7 and 9) adjusted using HCl and NaOH solutions and kept for 24 h at room temperature. After the passage of time, it was observed that the colour of the solution changed due to the formation of AgNPs. After 24 h, all the samples were centrifuged for 10 min at 12 000 rpm. The samples were washed thoroughly with ethanol, acetone and distilled water. The samples were transferred to Petri dishes and dried at 90 °C for 4 h on a water bath.

### 2.3 Characterization of the synthesized silver nanoparticles

UV-visible spectra of the samples were collected using a PerkinElmer double beam spectrophotometer (Lamda 35, Germany). The samples were scanned in the range of 350 nm to 500 nm.

X-ray diffraction (XRD) measurements were conducted on a Philips PW 1830 instrument diffractometer for investigation of the structure and other impurities. The tests were carried out at room temperature and between 20° to 80° of 2*θ*. In addition, the particle sizes were analyzed using the Scherrer equation (eqn (1)).^50^ The samples were produced by spreading the powder uniformly on to a quartz sample holder.1
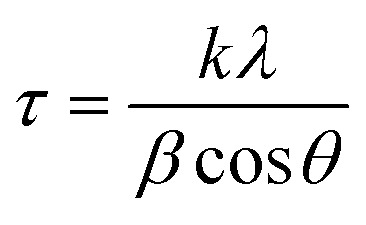
where *τ* = the average size of the nanoparticles, *k* = a dimensionless shape factor (value of 0.9), *λ* = the wavelength of radiation, *β* = full width half maximum in radians and *θ* = angle diffraction.

The surface characteristics and morphology were investigated using scanning electron microscopy (SEM) on a Phillips model CM 200 at 20 kV. The SEM test samples were carefully collected and screened in glass bottles. The SEM copper plate was covered by conductive resin tape and the particles were distributed on the tape and gold coated.

Atomic force microscopy AFM (Asylum MFP-3D-USA) was used for evaluating the particle size of the synthesized AgNPs. Sample preparation for AFM involved dilution of the sample in distilled water (ratio of 1 : 9). Two drops of the dilution were laid on the sample holder and allowed to dry in air.

The particle size and size distribution were obtained using a Malvern Zetasizer nano series instrument, UK, by dispersing them at a temperature of 25 °C in aqueous media.

The morphology of the prepared nanoparticles was investigated by TEM (JEM-2100, JEOL with an accelerating voltage of 200 kV, USA). Samples for the TEM analysis were produced by dispersion in ethanol solution through ultrasonication for 60 min and then centrifuged at 10 000 rpm for 10 min. After that, a few drops of the AgNPs were dropped on carbon-coated copper grids, then left to dry naturally.

FTIR spectrum analysis was performed to study the chemical constituents responsible for the reduction and capping agents of silver nanoparticles. A small amount of solid nanoparticles synthesized by NE and NOT extracts were mixed with KBr and a film was prepared and tested on a PerkinElmer spectrum 100 spectroscopy system (Waltham, MA, USA). The results were recorded in the range of 4000–500 cm^−1^.

### 2.4 Antibacterial activity

The Kirby disc diffusion method was used for determination for the antimicrobial tests. In order to analyze the response of the studied drugs with and without AgNPs against SA, the diameter of the inhibition zone was measured and was figured out for three responses; resistant, intermediate, and susceptible. The efficiency of the silver nanoparticles to cause cell rupture compared to silver ions is based on the cell type and is size-dependent. However, the exact mechanism by which silver nanoparticles cause an antimicrobial effect is still not clearly known. Although there are many approaches about their biocidal action on bacteria, AgNPs have the tendency to hold on to the cell wall of bacteria and later to penetrate it, causing structural changes in the cell membrane, such as in permeability, and afterward cell death.^51^ Silver is known to be a soft acid, and there is a natural potency of acids to react with bases; in this case, a soft acid reacts with the soft base. Cells are usually composed of phosphorus and sulfur, which are soft bases. Thus, a reaction between the nanoparticles' soft acid and cells' soft base takes place, which results in the formation of salt and also the death of cells takes place.^52^ The measurement of the diameter in mm according to the response of the drug was assigned a value, as detailed in Table 2. The definition of resistant, intermediate and susceptible responses under Kirby–Bauer antibiotic sensitivity is given in Table 3.

**Table 2:** Zone diameter interpreter chart (Becton, Dickinson and Company)

S. no.	Name of antibiotic	Resistant (Mm)	Intermediate (Mm)	Susceptible (Mm)
1	Amikacin	≤14	15–16	≥17
2	Amoxillian	≤13	14–17	≥18
3	Cefoaclor	≤14	15–17	≥18
4	Cefonicid	≤14	15–17	≥18
5	Clindamycin	≤14	15–17	≥18
6	Fosfomycin	≤12	13–15	≥16
7	Levofloxacin	≤13	14–16	≥17
8	Piperacillin	≤17	18–20	≥21

**Table 3:** Zone/region of inhibition (diameter) in mile meter

Response	Definition
Resistant	If bacteria are unaffected by an antibiotic with or without nanoparticles. In this situation, the inhibition zone diameter will be smaller. This means bacteria are not completely inhibited
Susceptible	If bacteria are positively affected by a specific antibiotic with or without silver nanoparticles. In this case, the inhibition zone diameter will be larger. This means the bacteria are completely inhibited
Intermediate	If bacteria are poorly affected by a specific antibiotic with or without silver nanoparticles. In this case, the inhibition zone diameter will be intermediate, since the growth of microorganisms is stopped to some extent by the medicine

#### 2.4.1 Procedure for the antibacterial tests

All glassware was carefully washed with distilled water. The nutrient solution was prepared by dissolving 10 g of nutrient agar in 50 ml of deionized water for microorganism cultivation. In order to accomplish a perfect mix, the mixture was heated a little at 30 °C to 40 °C for 10 min. Thereafter, the solution was transferred to an autoclave, where it was heated for a further 15 min at 121 °C for complete drying. SA microorganisms were introduced on a dried plate by using a cotton-tipped stick. Eight antibiotic medicine disks with a diameter of 6 mm and weight of 10 μg were introduced on a Petri dish at different spaces, and the synthesized NPs of different concentrations were then added. Later, the biosystems were left for 24 h at 37 °C for incubation. After 24 h, the zones of different diameters were measured and compared with the Kirby–Bauer graph to evaluate the susceptibility of the bacteria to the different antibiotics.

## 3. Results and discussion

### 3.1 UV-visible spectroscopy (UV-vis)

**Fig. 1:**
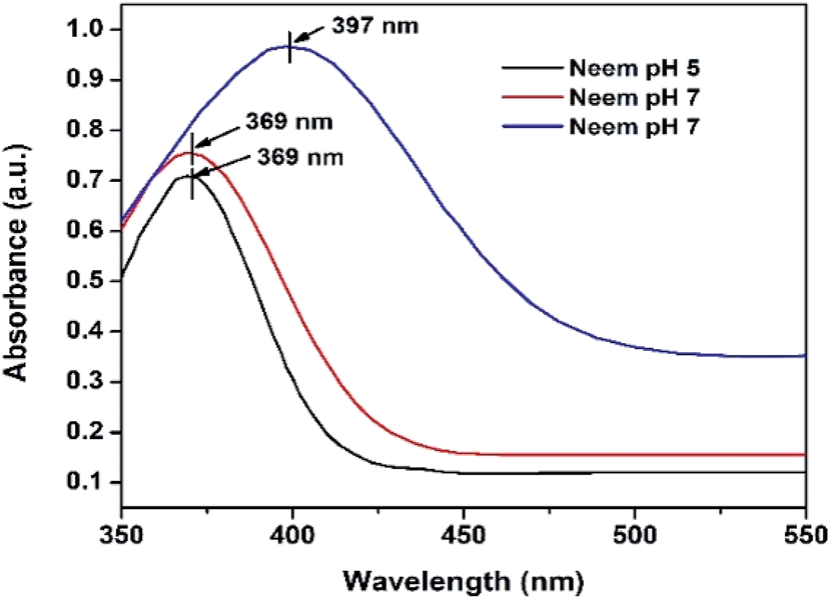
UV-vis of AgNPs using NE at (pH 5, pH 7, pH 9).

**Fig. 2:**
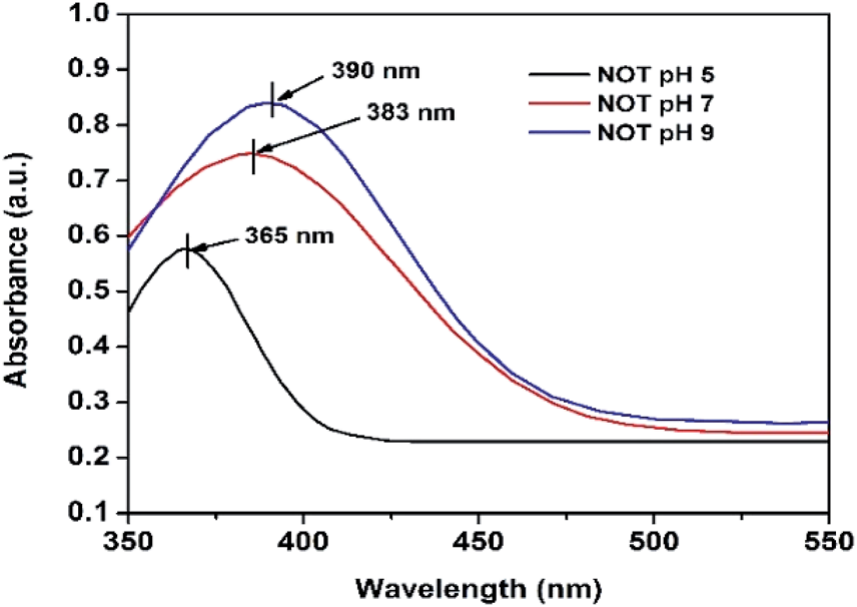
UV-vis of AgNPs using NOT mixed extract at (pH 5, 7 and 9).

The UV-vis analyses of the synthesized AgNPs at different pHs using NE and NOT extracts are shown in Fig. 1 and 2, respectively. Fig. 1 indicates that the AgNPs synthesized at pH 5 and 7 show an absorbance band at 369 nm; whereas for the AgNPs synthesized at pH 9, the bands were developed at 397 nm. In contrast, UV-vis bands observed in the AgNPs synthesized using NOT extract at pH 5, 7 and 9 were observed at 365, 383 and 390 nm, respectively, as shown in Fig. 2.

Theoretically, the variation in the bands corresponds to the variation of the colour, whilst the colour variation relates to the difference in the size of nanoparticles.^53,54^ The development of the bands indicates that silver ions present in the silver nitrate solution were successfully reduced to silver nanoparticles when exposed to the NE and NOT extracts. It is worth mentioning here that the absorption peaks of the AgNPs lie in the range of 360 to 396 nm, in accordance with the absorption peaks reported in the literature.^55–57^ These observations also confirmed that synthesized AgNPs of different particle sizes were formed with the different extracts.

### 3.2 X-ray diffraction (XRD)

**Fig. 3:**
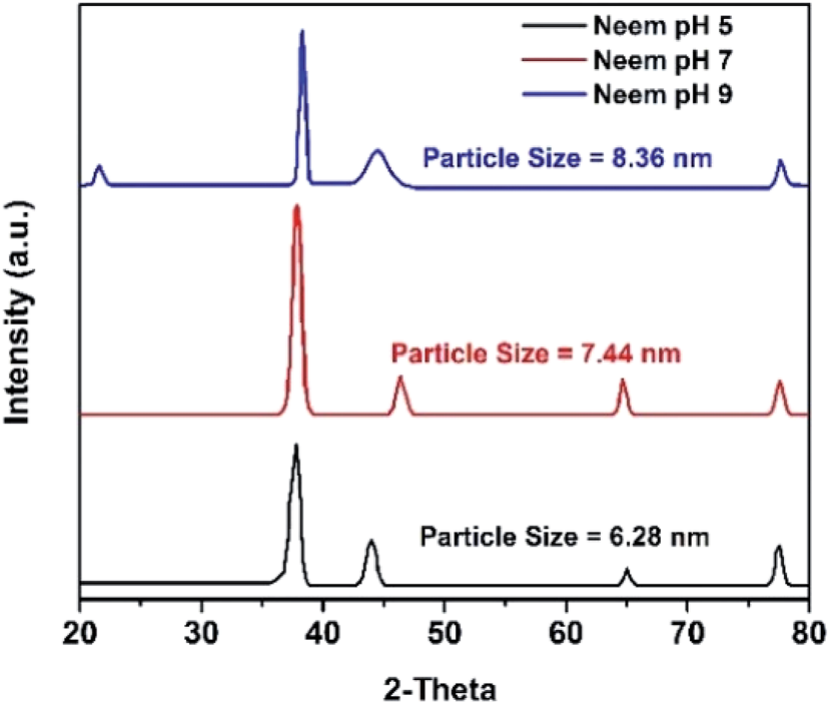
XRD results of AgNPs using NE (pH 5, 7, 9).

**Fig. 4:**
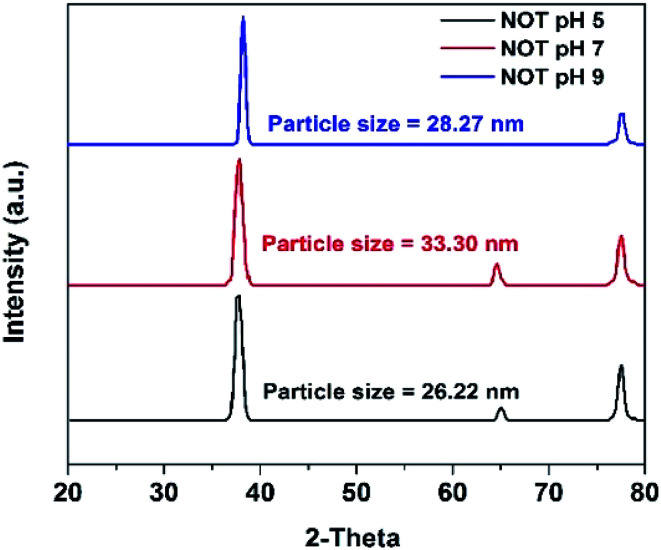
XRD results of AgNPs using NOT mixed extract (pH 5, 7, 9).

The XRD results of the AgNPs synthesized at different pHs using NE and NOT extracts are shown in Fig. 3 and 4, respectively. It can be seen in Fig. 3 and 4 that a prominent peak of AgNPs was developed in the range of 2*θ* (36.5° to 38°). The XRD pattern of AgNPs reported in the literature^21,30,58^ is inconsistent with the pattern shown in Fig. 3 and 4. Thus, using XRD, the development of AgNPs was confirmed. Interestingly, major effects of the pH and plant extract on the synthesis of AgNPs were observed related to the obtained size and structure, which meant that both extracts had a tendency to synthesize AgNPs with different natures.

The particle size of the synthesized AgNPs was calculated using *β*, deduced from the XRD patterns using Scherrer's equation (eqn (1)). The results are summarized in Table 4, and indicate that using NE, particles of 6.28, 7.44 and 8.36 nm were produced; whereas in the case of NOT extract, particles of 26.22, 33.3 and 28.27 nm were developed at pH 5, 7 and 9, respectively. From this, it was noted that NOT extract produced coarser AgNPs as compared to NE.

**Table 4:** Particle size analysis of synthesized AgNPs by using various techniques

Sample name	pH	XRD			AFM	DLS		TEM
		FWHM (Deg)	2*θ* (Deg.)	Particle size (nm)	Size (nm)	Size range (nm)	PDI	Size range (nm)
NE	5	0.662	38.15	6.28	9	6–28	0.289	6
	7	0.562	37.95	7.44	2	3–28	0.389	7.66
	9	0.496	38.305	8.36	2	3–10	0.486	16.8
NOT	5	0.159	38.1	26.22	25	15–500	0.244	13
	7	0.125	38.15	33.30	30	15–600	0.812	17.4
	9	0.148	37.95	28.27	20	10–800	1.00	36

### 3.3 Atomic force microscopy (AFM)

**Fig. 5:**
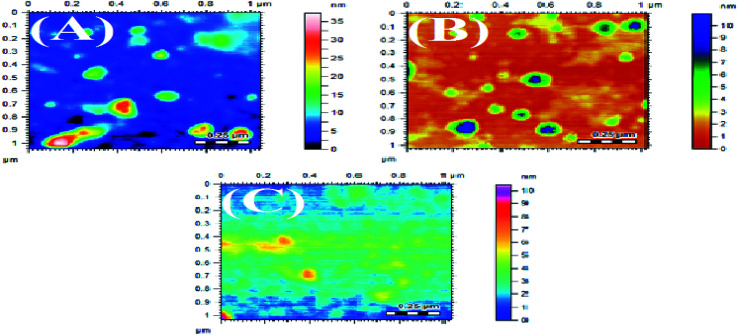
AFM images of AgNPs using NE at pH 5 (A), pH 7 (B), pH 9 (C).

**Fig. 6:**
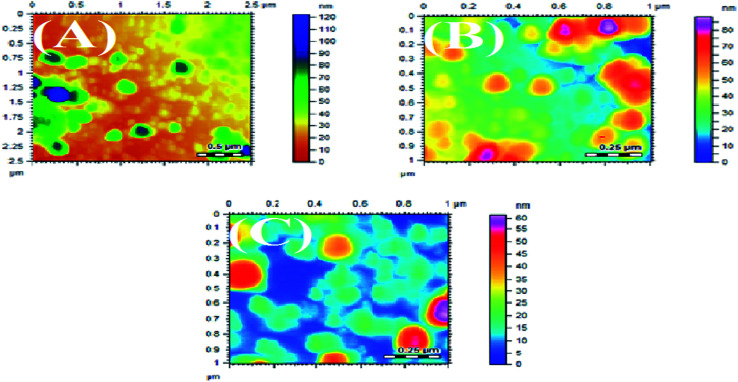
AFM images of AgNPs using NOT at pH 5 (A), pH 7 (B), pH 9 (C).

The synthesized AgNPs were also studied using AFM. The AFM images are shown in Fig. 5 and 6, synthesized at different pHs. The average size range of AgNPs deduced from the AFM images is given in Table 4. It can be substantiated from the AFM results given in Table 4 that the AgNPs produced using NE were finer than the particles developed with the NOT extract.^5,59^ The effect of pH on the obtained particle size was consistent with the results obtained by XRD.

### 3.4 DLS technique

**Fig. 7:**
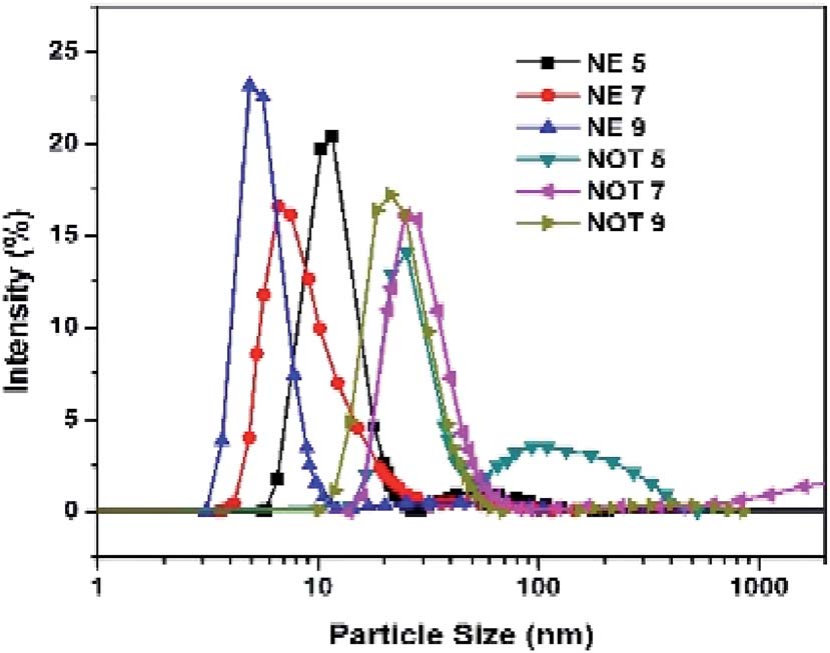
Size distribution of AgNPs using NE and NOT at (pH 5, 7, 9).

The size and size distribution of the synthesized AgNPs obtained using the Zetasizer after dispersion in water at a temperature of 25 °C are shown in Fig. 7, and tabulated in Table 4. The DLS curves indicated that particles of different sizes could be obtained by varying the pH of the solution. From the value of PDI, it is evident that the NOT extract produced AgNPs with a wide size distribution as compared to NE.

The effect of the pH of all the extracts also suggested that the particle size values were lower for an acidic medium and higher for a basic medium. It is always the case that size distribution evaluated through DLS is better than from UV-vis spectroscopy.^60^

### 3.5 Scanning electron microscopy (SEM)

**Fig. 8:**
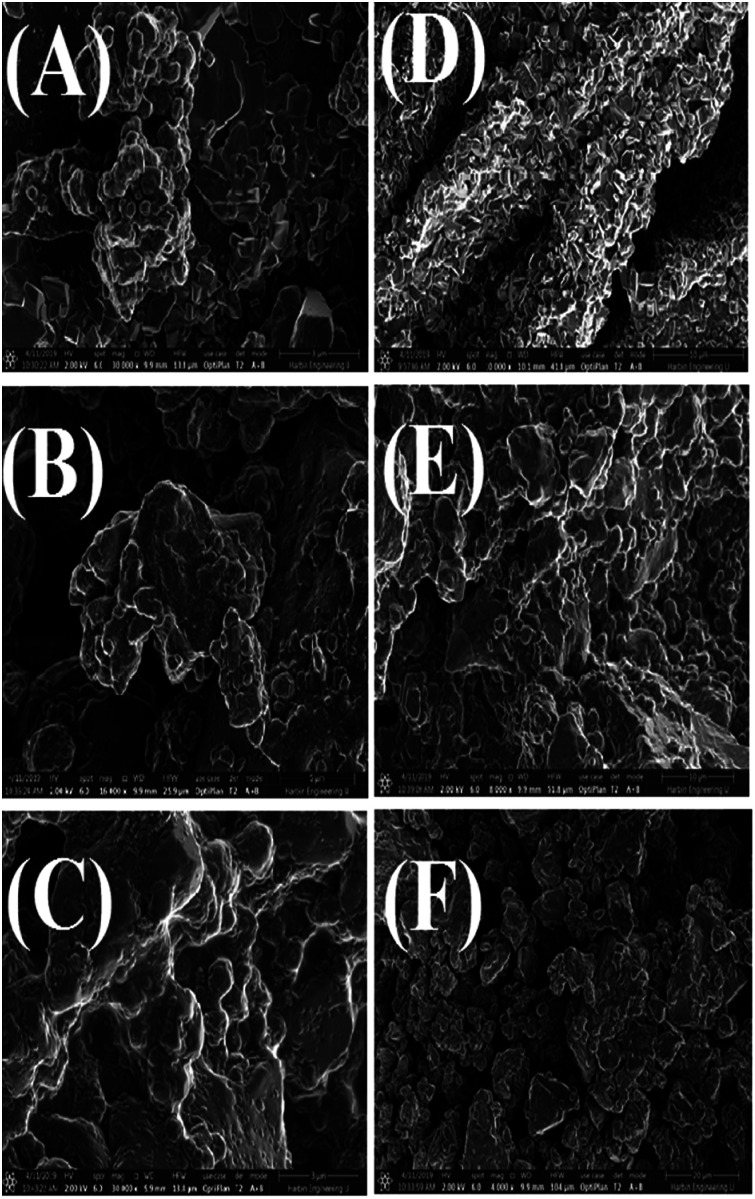
SEM images of AgNPs using NE pH 5 (A), pH 7 (B), pH 9 (C) and NOT pH 5 (D), pH 7 (E), pH 9 (F).

SEM images of the AgNPs synthesized using NE and NOT extracts at different pH values are shown in Fig. 8. It can be seen from Fig. 8 that AgNPs with a round texture and spherical shape with mild agglomeration and lumps were developed. It is reported that nanoparticles have a great potential to aggregate in solution. For drug delivery and antibacterial studies, the interaction potential of nanoparticles with cells is based on gravitation, diffusion and convection forces.^61,62^ More agglomerated particles show less effect on the cellular level. The aggregation process might be affected by the pH and protein composition in the culture medium. According to^63^ 20–200 nm-sized AgNPs aggregated in the culture medium, the aggregation range changed depending on the NPs solution. The hydrodynamic diameter of the AgNPs could be larger than the nominal size of the particles. Numerous studies have revealed that the binding capacity of NPs with protein is different and is based on the NPs and the chemical structure of the protein.

### 3.6 Fourier-transform infrared spectroscopy (FTIR)

**Fig. 9:**
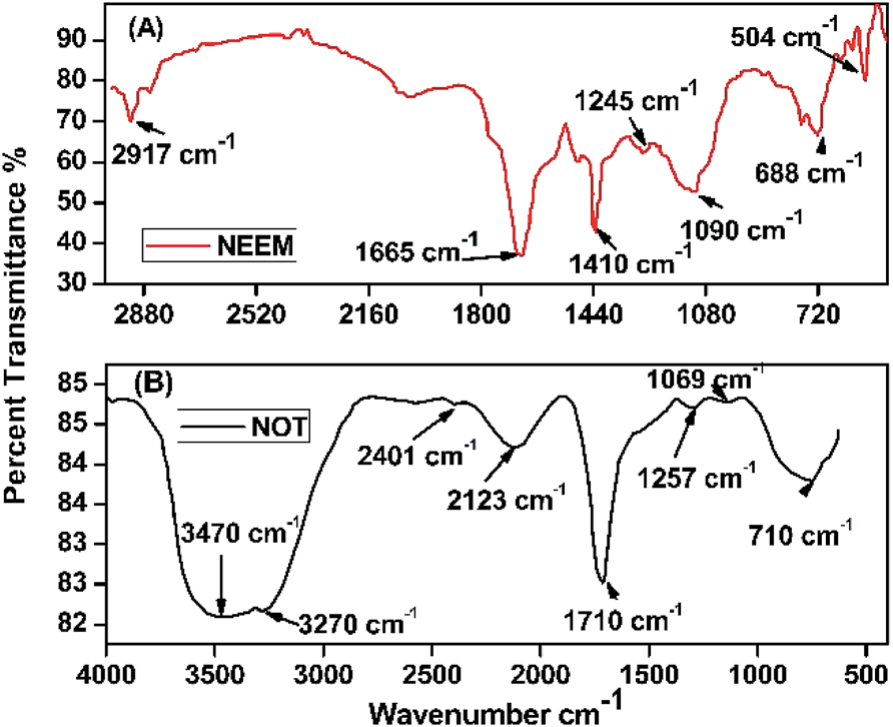
FTIR spectra of AgNPs using (A) neem and (B) NOT.

FTIR analysis of the AgNPs synthesized using NE and NOT extracts are shown in Fig. 9. The FTIR spectrum in the range between 3500–3200 cm^−1^ represents O–H stretching and H-bonded alcohols and phenols. The peak found around 2260–2100 cm^−1^ shows a stretch for (–C=C–) bond, which corresponds to alkenes, while the peak found around 1650–1580 cm^−1^ shows the bond for (N–H) bending, which corresponds to primary amines, and peak around 690–500 cm^−1^ shows the bond for (–C=C–H:C·H) bending, which again corresponds to alkenes, and the bends between 1700–1400 cm^−1^ are mainly responsible for the formation of silver nanoparticles.^9,64^

Plant extracts play a dual nature role: as a reducing agent and stabilizing agent. Furthermore, the presence of many functional groups was confirmed by FTIR analysis. The reduction of silver nanoparticles was accomplished due to the phenolics, terpenoids, polysaccharides and flavones compounds present in the extract.^65^ Flavonoid and terpenoid compounds present in the extract were claimed to be responsible for the stabilization of nanoparticles.^7^ Possible chemical constituents of the plant extracts are responsible for the bioreduction of metal ions.^65,66^

### 3.7 Transmission electron microscopy (TEM)

**Fig. 10:**
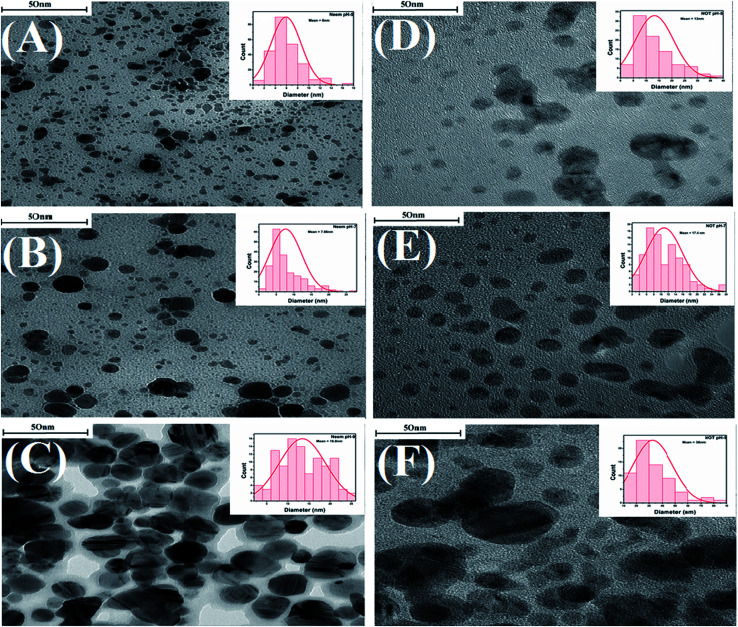
TEM images of AgNPs synthesized with neem at various pH, 5 (A), 7 (B) and 9 (C); NOT at various pH 5 (D), 7 (E) and 9 (F), the internal image shows the histogram for the particle-size distribution.

Characterization of the AgNPs was also carried out by using TEM. TEM is one of the most popular techniques used for the identification of the shape, size and morphology of nanoparticles synthesized by various methods, including green synthesis.^67,68^ The TEM images with the particles size distributions displayed as insets in the form of histogram charts are portrayed in Fig. 10.

Fig. 10 shows that as the pH increased from 5 to 7, the average particle sizes also increased. The AgNPs synthesized with neem extracts at pH 5, 7, and 9 showed average particle sizes of 6, 7.66 and 16.8 nm, respectively (Fig. 10A–C). Similarly, the corresponding average particle sizes for the AgNPs synthesized with NOT extracts were recorded as 13, 17.4 and 36 nm, respectively (Fig. 10D–F). The average size range of AgNPs deduced from the TEM images is also given in Table 4. A previous study was conducted by Verma *et al.* and reported that the particle size of AgNPs could be controlled by varying the pH of solution.^9^ These results are in a good agreement with the aforementioned UV-vis, XRD, DLS and AFM results.

The TEM images in Fig. 10 show that all of the synthesized AgNPs made with both neem and NOT extracts were in the nanometric size (up to 100 nm) with spherical, irregular morphologies and a polydispersed character. In addition, the findings showed that the AgNPs synthesized with NOT extracts were larger compared to the neem extracts, due to the flavonoid and terpenoid compounds presents in the NOT extracts.

### 3.8 Antibacterial tests

**Fig. 11:**
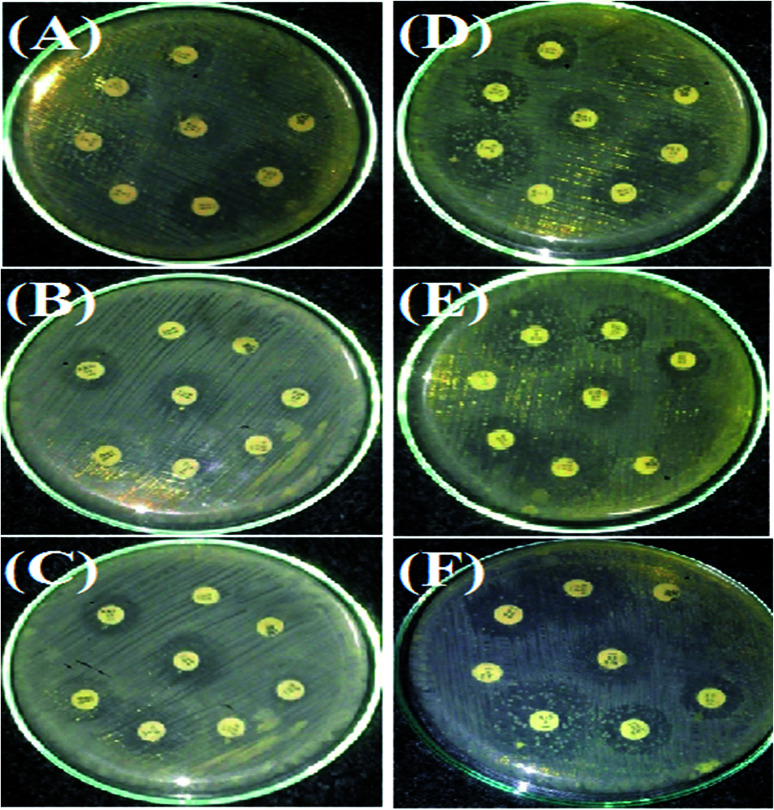
Antimicrobial tests for SA microorganism by AgNPs in a culture using Kirby disc diffusion. NE (A–C) and NOT (D–F).

**Fig. 12:**
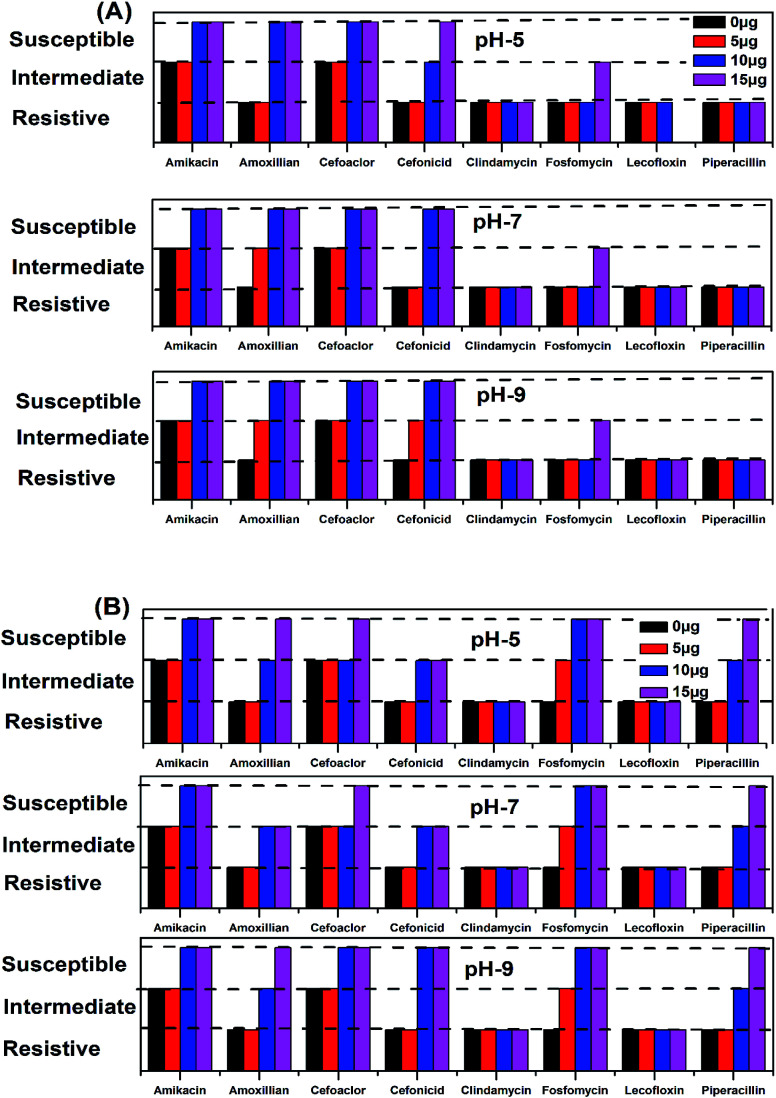
Antimicrobial activity zone/region of inhibition of AgNPs for different classes of medicines, using NE (A) and NOT (B).

The antimicrobial action of all the synthesized AgNPs was investigated by using the culture of SA microorganism using the Kirby disc diffusion method, as shown in Fig. 11. Fig. 9A–C show the antibacterial effects of the NE-synthesized and Fig. 11D–F the NOT-synthesized AgNPs at pH 5, 7 and 9, respectively. The antibacterial activity value obtained using different μg of synthesized AgNPs of all the products against SA using NE and NOT are shown in Fig. 11 and 12, while the values are tabulated in Tables 5 and 6.

**Table 5:** Antibacterial activity of AgNPs using NE (pH 5, 7, 9)

S. no.	Name of antibiotic	pH 5								pH 7						pH 9					
		0 μg		5 μg		10 μg		15 μg		5 μg		10 μg		15 μg		5 μg		10 μg		15 μg	
1	Amikacin	16	I	16	I	18	S	19	S	16	I	19	S	20	S	16	I	19	S	20	S
2	Amoxillian	13	R	13	R	19	S	19	S	14	I	18	S	18	S	14	I	18	S	18	S
3	Cefoaclor	17	I	17	I	20	S	21	S	17	I	18	S	18	S	17	I	19	S	19	S
4	Cefonicid	14	R	15	R	16	I	18	S	15	R	18	S	19	S	15	I	18	S	18	S
5	Clindamycin	10	R	10	R	10	R	10	R	10	R	10	R	10	R	10	R	10	R	10	R
6	Fosfomycin	12	R	12	R	12	R	13	I	12	R	12	R	13	I	13	R	13	R	14	I
7	Lecofloxin	12	R	12	R	12	R	12	R	12	R	12	R	12	R	12	R	12	R	12	R
8	Piperacillin	10	R	12	R	14	R	14	R	12	R	13	R	14	R	12	R	12	R	13	R
Total	Resistive	6		6		3		3		5		4		3		4		4		3	
	Intermediate	2		2		1		1		3		0		1		4		0		1	
	Susceptible	0		0		4		4		0		4		4		0		4		4	

**Table 6:** Antibacterial activity of AgNPs using NOT (pH 5, 7, 9)

S. no.	Name of antibiotic		pH 5								pH 7						pH 9					
			0 μg		5 μg		10 μg		15 μg		5 μg		10 μg		15 μg		5 μg		10 μg		15 μg	
1	Amikacin		16	I	16	I	18	S	19	S	16	I	18	S	19	S	16	I	18	S	18	S
2	Amoxillian		13	R	13	R	17	I	19	S	14	R	16	S	19	S	13	R	16	I	19	S
3	Cefoaclor		17	I	17	I	17	I	18	S	17	I	17	I	18	S	17	I	18	S	20	S
4	Cefonicid		14	R	13	R	18	S	22	S	14	R	19	S	20	S	14	R	18	S	21	S
5	Clindamycin		10	R	11	R	14	R	12	R	11	R	12	R	13	R	12	R	12	R	13	R
6	Fosfomycin		12	R	15	I	17	S	25	S	15	I	24	S	25	S	16	I	25	S	25	S
7	Lecofloxin		12	R	13	R	13	R	14	R	13	R	0	R	13	R	12	R	13	R	13	R
8	Piperacillin		10	R	13	R	17	I	22	S	13	R	18	I	20	S	12	R	19	I	23	S
Total		Resistive	6		5		2		2		5		2		2		5		2		2	
		Intermediate	2		3		3		0		3		2		0		3		2		0	
		Susceptible	0		0		3		6		0		4		6		0		4		6	

All the medicines without the addition of AgNPs showed resistance to SA, except Amikacin and Cefoaclor, which showed intermediate antibacterial activity. For the products synthesized at pH 5, the addition of 5 μg of NE product caused no change, while the NOT product changed the resistivity of Fosfomycin to the intermediate level. At a higher concentration of AgNPs addition, different behaviour on different medicines was observed. At 10 μg addition of the NE product AgNPs, the range of Amikacin, Amoxillian and Cefoaclor changed to susceptible, and Cefonicid to intermediate. However, for the same amount of NOT product, the resistivity of Amikacin, Cefonicid and Fosfomycin increased to susceptible and Amoxillian, Cefoaclor and Piperacillin to intermediate. Similar behaviour was also observed for the higher concentration of 15 μg, whereupon the NE product changed Fosfomycin to intermediate and Cefonicid to a susceptible range, while the NOT product changed Amoxillian, Cefoaclor and Piperacillin to susceptible values.

For the products synthesized at pH 7, the 5 μg addition of NE product showed the resistivity of SA for Amoxillian, while the NOT product showed the resistivity of Fosfomycin. The resistivity of both changed to an intermediate level from resistive. At higher concentration, 10 μg of NE product changed Amikacin, Amoxillian and Cefoaclor from intermediate to susceptible and Cefonicid from resistive to susceptible. For the NOT product at the same concentration, Amikacin and Cefoaclor changed behaviour from intermediate to susceptible, while Amoxillian, Cefonicid and Piperacillin changed from resistive to intermediate. A major improvement was observed for Fosfomycin, which changed from resistive to completely susceptible. At 15 μg of AgNPs addition, the NE product showed intermediate activity from resistive, while the NOT product changed Cefoaclor and Piperacillin from intermediate to completely susceptible.

The medicines also showed improvements in antibacterial activity against SA for the products synthesized at pH 9. Here, 5 μg addition of NE product changed Cefonicid and Amoxillian to an intermediate level. However, only Fosfomycin was affected by the NOT product, which also showed an intermediate value. At 10 μg addition of synthesized AgNPs, the NE product increased the antibacterial effect of Amikacin, Amoxillian, Cefoaclor and Cefonicid to susceptible, while the NOT product change Amikacin, Cefoaclor, Cefonicid and Fosfomycin to susceptible and Amoxillian and Piperacillin to intermediate. Upon the further addition of AgNPs, *i.e.* 15 μg, the NE product only affected Fosfomycin, while the NOT product increased the antimicrobial activity of Amoxillian and Piperacillin from intermediate to completely susceptible.

In the summary of the antimicrobial activity of all the synthesized products, it was observed that AgNPs synthesized with NE and NOT products worked with four and six medicines, respectively, at the amount of 15 μg. In both cases, the addition of 10 μg was found to be sufficient to change the zone to susceptible, which proved that the synthesized AgNPs using NE and NOT worked well for achieving antimicrobial activity against SA microorganisms. However, better results were achieved for the products of NOT. This may be due to the different nature of the functional groups (flavonoids and tripenoids) present in the mixture of NOT.

## 4. Conclusions

The synthesis of AgNPs through a green route using NE and NOT extracts at pH 5, 7 and 9 was successfully performed and studied. UV-vis spectroscopy confirmed that the wavelength lies in the range of 360 to 396 nm. XRD results showed the existence of 2*θ* peaks at 38°, 44°, 65° and 78° in the spectra, which reflected that the AgNPs crystal size and structure was dependent on the pH of the extraction medium. Besides this, AFM, SEM and TEM images verified that the AgNPs synthesized with NE extracts had smaller particle size than those made from the NOT extracts and the NE particles had a looser structure than NOT, respectively. As a whole, particles with a round texture and spherical shape with mild agglomeration and lumps were obtained. Furthermore, the FTIR results proved that flavonoid and tripenoid compounds were acting as reducing and capping agents. The antibacterial activity of AgNPs products were performed against SA and it was observed that the results of the NOT products were improved over the NE products. The results, though, were contradictory, as for instance, the particle size and structure of AgNPs products obtained with NE extracts were small and loose as observed in the characterization techniques; however, the reason for this could be attributed to the different nature of flavonoid and terpenoid compounds in the NOT extracts. Another reason could be the greater aggregation of the smaller sized particles supplemented with medicines in the nutrient ager treatment conditions. This study opens new ways to perform the green synthesis of AgNPs with a NOT mixture with improved antimicrobial activity against SA.

## Conflicts of interest

The authors declare no conflict of interest.

## Supplementary Material
